# Hierarchical ordering with partial pairwise hierarchical relationships on the macaque brain data sets

**DOI:** 10.1371/journal.pone.0177373

**Published:** 2017-05-18

**Authors:** Woosang Lim, Jungsoo Lee, Yongsub Lim, Doo-Hwan Bae, Haesun Park, Dae-Shik Kim, Kyomin Jung

**Affiliations:** 1 School of Computing, KAIST, Daejeon, Korea; 2 School of Electrical Engineering, KAIST, Daejeon, Korea; 3 Department of Computer Science and Engineering, Seoul National University, Seoul, Korea; 4 School of Computational Science and Engineering, Georgia Tech, Atlanta, United States of America; 5 Department of Electrical and Computer Engineering, Seoul National University, Seoul, Korea; Banner Alzheimer’s Institute, UNITED STATES

## Abstract

Hierarchical organizations of information processing in the brain networks have been known to exist and widely studied. To find proper hierarchical structures in the macaque brain, the traditional methods need the entire pairwise hierarchical relationships between cortical areas. In this paper, we present a new method that discovers hierarchical structures of macaque brain networks by using partial information of pairwise hierarchical relationships. Our method uses a graph-based manifold learning to exploit inherent relationship, and computes pseudo distances of hierarchical levels for every pair of cortical areas. Then, we compute hierarchy levels of all cortical areas by minimizing the sum of squared hierarchical distance errors with the hierarchical information of few cortical areas. We evaluate our method on the macaque brain data sets whose true hierarchical levels are known as the FV91 model. The experimental results show that hierarchy levels computed by our method are similar to the FV91 model, and its errors are much smaller than the errors of hierarchical clustering approaches.

## Introduction

Hierarchical organization in the brain networks has been known to enable the efficient processing of information to support complex brain functions, and it has been studied in various ways to understand structural and functional brain networks [1, 2]. Among the recent studies, most of the work can be roughly categorized into the following two types of approaches: finding hierarchical modularity in the brain [3–7], and hierarchical ordering of cortical areas in the brain [8–14]. The former computes a hierarchy of modules by partitioning the organization into submodules without using pairwise hierarchical relationships, but the result may not reflect the information flow in the brain. Whereas the latter computes hierarchy levels of cortical areas which successfully reflect the information flow, but it needs pairwise hierarchical relationships.

Hierarchical ordering of cortical areas in the brain was proposed to provide the understanding and insight about cortical structure and function by Felleman and Van Essen (1991) [8]. To compute hierarchy orders of cortical areas, they first obtained the connectivities of cortical areas which imply the existence of information flow between two cortical areas from tract tracing experiments. Then, they derived 305 pairwise hierarchical relationships for the 32 cortical areas by observing differential laminar source and termination patterns, and the pairwise hierarchical relationships consists of three types on the pair of cortical areas which have a connection between them: feedforward, feedback, and lateral. Specifically, the lateral pairwise hierarchical relationship corresponds to the flow of information between two cortical areas whose hierarchy levels are the same. The feedforward pairwise hierarchical relationship corresponds to the flow of information from a cortical area of lower hierarchy level to a cortical area of higher hierarchy level, and the reverse direction flow corresponds the feedback. Especially, feedforward has two different types of weights which are ascending and strongly ascending, and feedback also has two different types of weights which are descending and strongly descending [8, 12]. Based on these extracted pairwise hierarchical relationships, Felleman and Van Essen proposed the FV91 model which describes 10 discrete hierarchy levels of information processing and a global view of areal relations (Table 1) [8]. Similarly, other related studies also compute hierarchy levels based on the entire pairwise hierarchical relationships [9, 12, 14–17].

**Table 1:** The FV91 model: Hierarchy levels of macaque vision and somatosensory-motor data sets [8].

Hierarchy Level	The FV91 Model (vision)	The FV91 Model (somatosensory-motor)
9	46 TF TH	35 36
8	STPa AITd AITv	SMA 6 ld
7	7a FEF STPp CITd CITv	4 lg
6	VIP LIP MSTd MSTI FST PITd PITv	7a 7b
5	DP VOT	Sll
4	MDP MIP PO MT V4t V4	Rl
3	PIP V3A	5
2	V3 VP	2
1	V2	1
0	V1	3a 3b

Although traditional methods including the FV91 model successfully compute hierarchical orders of cortical areas in the brain, they need almost the entire pairwise hierarchical relationships to compute hierarchy levels, and the technique of obtaining pairwise hierarchical relationships is complicated. For example, the technique using invasive method and anatomical criteria is not applicable to the in vivo human brain, thus there is a very limited amount of pairwise hierarchical information for human brain. Consequently, we have a question; How can we compute hierarchy orders of cortical areas by using a restricted amount of pairwise hierarchical relationships with small errors?

In this paper, we propose a novel method which computes hierarchy orders with the connectivities of cortical areas and a smaller amount of pairwise hierarchical relationships. More specifically, since the connectivities of cortical areas do not imply the pairwise hierarchical relationships between cortical areas, we use the pseudo hierarchical distance to overcome the lack of pairwise hierarchical relationships. Although we use a restricted amount of pairwise hierarchical relationships, our method computes the hierarchy levels of cortical areas with small errors. Whereas the traditional approaches need the entire pairwise hierarchical relationships of cortical areas to compute accurate hierarchy levels. In the later sections, we will provide the motivation and the detailed description of our method. We will also evaluate our method on the macaque brain data sets, and compare the experimental results with the variants of FV91 models using the entire pairwise hierarchical relationships and the results of hierarchical clustering approaches.

## Methods

In this section, we propose a novel method that can solve the hierarchical ordering problem by using the connectivities of cortical areas and the partial information of their pairwise hierarchical relationships. We note that the connectivities of cortical areas corresponds to the existences of information flow between two cortical areas, and do not imply the pairwise hierarchical relationships between cortical areas. Thus, we define *pseudo hierarchical distance* to overcome the lack of pairwise hierarchical relationships, and compute hierarchy levels of cortical areas by minimizing the hierarchical distance errors. Our method is displayed in Alg 1, and it consists of three parts: manifold learning, modeling the hierarchical distance, minimizing the hierarchical distance errors. We will discuss them in details in the following sections.

1. **Manifold Learning.** In this part, we compute the *k*-dimensional embedding of cortical areas in the brain by using connectivity of cortical areas without the pairwise hierarchical relationships, so that we can capture the unseen relationships between cortical areas by using *k*-dimensional vectors. Especially, we use Laplacian eigenmap to compute *k*-dimensional representative vectors given the adjacency matrix of brain connectivity graph.
2. **Modeling Hierarchical Distance.** We assume that if the information is processed in stepwise fashion, then the direct interaction between two cortical areas which have a large hierarchical distance would be relatively rare. Based on this assumption, in this part we compute similarities *s*(*u*_*i*_, *u*_*j*_) for every pair of nodes by using *k*-dimensional embedding, and define pseudo hierarchical distance *μ*(*u*_*i*_, *u*_*j*_) by using similarities *s*(*u*_*i*_, *u*_*j*_) to overcome the lack of pairwise hierarchical relationships.
3. **Minimizing the Hierarchical Distances Errors.** In this part, we set the hierarchy level *h*(*u*_*i*_) for a few nodes, e.g., *h*(V1) = 0, and compute hierarchy levels of cortical areas by minimizing the sum of the square of the hierarchical distance errors for all the pairs.

**Algorithm 1** Hierarchical Ordering with Partial Pairwise Hierarchical Relationships

**Input:** The *n* × *n* weighted adjacency matrix **W**

**Output:** Hierarchy levels **h** = (*h*(*u*_1_), …, *h*(*u*_*n*_)) for *n* cortical areas

1: **Manifold Learning.**

 Eigen-decompose a directed graph Laplacian **L**_*d*_ defined in Eq (1) with rank-*k* and contruct **Y** in Eq (2)

 Compute **Z** in Eq (3) by normalizing row vectors of **Y**

2: **Modeling Hierarchical Distance.**

 Compute a regularized Tanimoto similarity *s*(*u*_*i*_, *u*_*j*_) for every pair by using **Z** (Eq (4))

 Compute a pseudo hierarchical distance *μ*(*u*_*i*_, *u*_*j*_) by using *s*(*u*_*i*_, *u*_*j*_) (Eq (5))

3: **Minimizing the Error Function.**

 Fix the hierarchy level for some cortical areas, *e.g.*, *h*(*u*_*i*_) = 0

 Compute **h** = (*h*(*u*_1_), …, *h*(*u*_*n*_)) by minimizing ∑_*u*_*i*_,*u*_*j*__(|*h*(*u*_*i*_) − *h*(*u*_*j*_)| − *μ*(*u*_*i*_, *u*_*j*_))^2^ in the interval [0, *K*]

 Map *h*(*u*_*i*_) into discrete domain {0, 1, …, *K*}

### Manifold learning based on directed normalized Laplacian

Before computing similarities between cortical areas, we first use manifold learning to compute *k*-dimensional vectors which represent cortical areas in the brain. To computes low-dimensional representative vectors, we use graph Laplacian eigenmap which is one of the manifold learning techniques, and we will introduce it in this section. We note that we do not use pairwise hierarchical relationships to compute such low-dimensional embedding, and we only need connectivity matrix of cortical areas whose weight elements are 0 or 1, where the positive weight 1 implies the existences of information flow between two cortical areas.

Suppose that two cortical areas are connected or they have relatively many common connected cortical areas. Then, we argue that their *k*-dimensional representative vectors should be close to each other. Manifold learning is suitable to compute such representative vectors, since it computes a low-dimensional representation of the data set which preserves locality properties [18, 19]. That is, if two points **x**_1_ and **x**_2_ are similar or close to each other, then **f**(**x**_1_) and **f**(**x**_2_) are close to each other, where **f**(**x**) = (*f*_1_(**x**), …, *f*_*k*_(**x**)) is a *k*-dimensional representative vector computed by the manifold learning given the data **x**.

Laplacian eigenmap is one of the effective manifold learning techniques given the graph *G* = (*V*, *E*), and it is widely used in data mining and machine learning, where *n* = |*V*| [18, 20–22]. Among its variants, combinatorial Laplacian **L** = (**D** − **W**) is the most basic Laplacian operator given the symmetric graph, where **W** is the *n* × *n* adjacency matrix such that its element **W**_*i*,*j*_ is 1 when there exist an edge from node *u*_*i*_ to *u*_*j*_ and zero when there is no edge, and **D** is the diagonal matrix consisting of *d*(*u*_*i*_) = ∑_*j*_ **W**_*i*,*j*_. We can compute *k*-dimensional representative vectors by computing the first *k* eigenfunctions of Laplacian operator **L**. There are also normalized Laplacian matrices of symmetric graph which are **L**_*rw*_ = **I** − **D**^−1^ **W** and $L_{sym}=I-D^{-\frac{1}{2}}WD^{-\frac{1}{2}}$. Since the brain networks are directed, we use directed normalized Laplacian which reflects information flows to compute *k*-dimensional representative vectors.

Normalized directed Laplacian **L**_*d*_ is defined as

$$\begin{array}{c} L_{d}=I-\frac{\Psi^{1/2}P\Psi^{-1/2}+\Psi^{-1/2}P^{⊤}\Psi^{1/2}}{2}, \end{array}$$ (1)

where the Perron vector ***ψ*** is the unique left eigenvector corresponding the largest eigenvalue *ρ* of the transition matrix **P** = **D**^−1^ **W**, and **Ψ** is a *n* × *n* diagonal matrix with **Ψ**_*ii*_ = ***ψ***_*i*_. Let **Y** be the matrix which consists of the first *k* eigenvectors of **L**_*d*_, then row vectors of **Y** are *k*-dimensional vectors

$$\begin{array}{c} Y=\left[y_{1},...,y_{k}\right]\;\;\text{subject}\;\text{to}\;\;L_{d}y_{i}=\lambda_{i}y_{i}, \end{array}$$ (2)

where eigenvalues of **L**_*d*_ satisfy 0 = *λ*_1_ < *λ*_2_ ≤ … ≤ *λ*_*n*−1_, and **y**_*i*_ is the first *i*-th eigenvector of **L**_*d*_. Especially, we normalize row vectors of **Y** so that each row vector lies on the surface of *k*-dimensional sphere as

$$\begin{array}{c} Z={\left(\text{diag}\left(Y^{⊤}Y\right)\right)}^{-1}Y. \end{array}$$ (3)

That is, the row vectors of **Z** in Eq (3) are *k*-dimensional representative vectors of cortical areas in the brain.

### Modeling the hierarchical distance

In this section, we compute similarities between cortical areas by using *k*-dimensional representative vectors, and model the hierarchical distance to overcome the lack of pairwise hierarchical relationships.

We use Tanimoto similarity and *k*-dimensional representative vectors to compute similarities between cortical areas. Tanimoto Similarity is a generalized version of Jaccard similarity, which measures the similarities between vectors [23]. For two vectors **f**(*u*_*i*_) and **f**(*u*_*j*_), it is defined as $\frac{f{\left(u_{i}\right)}^{⊤}f\left(u_{j}\right)}{∥f\left(u_{i}\right)-f\left(u_{j}\right){∥}_{2}^{2}+f{\left(u_{i}\right)}^{⊤}f\left(u_{j}\right)}$. To limit the maximum and minimum values, we define a similarity *s*(*u*_*i*_, *u*_*j*_) of two cortical areas *u*_*i*_ and *u*_*j*_ by using regularized Tanimoto similarity

$$\begin{array}{c} s\left(u_{i},u_{j}\right)=\frac{f{\left(u_{i}\right)}^{⊤}f\left(u_{j}\right)}{∥f\left(u_{i}\right)-f\left(u_{j}\right){∥}_{2}^{2}+f{\left(u_{i}\right)}^{⊤}f\left(u_{j}\right)+\epsilon }, \end{array}$$ (4)

where *ϵ* = 1/*δ* and *s*(*u*_*i*_, *u*_*j*_) ∈ [−*δ*, *δ*].

Now we model the hierarchical distance of every pair based on the similarity *s*(*u*_*i*_, *u*_*j*_) defined in Eq (4). Let $h(u_{i})\in R$ be a hierarchical level of cortical area *u*_*i*_, and $z(u_{i},u_{j})\in R$ be an absolute value of difference between hierarchical levels of cortical areas *u*_*i*_ and *u*_*j*_, i.e., *z*(*u*_*i*_, *u*_*j*_) = |*h*(*u*_*i*_) − *h*(*u*_*j*_)|. If we set max_*u*_*i*__ *h*(*u*_*i*_) = *K* and min_*u*_*i*__ *h*(*u*_*i*_) = 0, then *h*(*u*_*i*_) ∈ [0, *K*] and *z*(*u*_*i*_, *u*_*j*_) ∈ [0, *K*]. Since we do not know the exact *z*(*u*_*i*_, *u*_*j*_), we introduce a pseudo hierarchical distance *μ*(*u*_*i*_, *u*_*j*_) which is an estimated mean of *z*(*u*_*i*_, *u*_*j*_). We define *μ*(*u*_*i*_, *u*_*j*_) as

$$\begin{array}{c} \mu \left(u_{i},u_{j}\right)=\delta -s\left(u_{i},u_{j}\right), \end{array}$$ (5)

where *δ* = *K*/2 and *ϵ* = 1/*δ*. We note that the pseudo hierarchical distance *μ*(*u*_*i*_, *u*_*j*_) is in the interval [0, *K*]. The pseudo hierarchical distance *μ*(*u*_*i*_, *u*_*j*_) between cortical areas *u*_*i*_ and *u*_*j*_ is small when their similarity *s*(*u*_*i*_, *u*_*j*_) is large, and *μ*(*u*_*i*_, *u*_*j*_) is large when their similarity *s*(*u*_*i*_, *u*_*j*_) is small.

### Minimizing hierarchical distance errors

In this section, we define the objective function based on the pseudo hierarchical distance, and compute hierarchy levels of all cortical areas by minimizing the objective function.

We note that the hierarchical distances in the graph are symmetric and nonnegative, even if the graph is directed. Thus, we consider a symmetric objective function, whereas we used the directed normalized Laplacian in the previous sections. Basically, we want to minimize the sum of the squared error of *z*(*u*_*i*_, *u*_*j*_) and *μ*(*u*_*i*_, *u*_*j*_) for all pairs. However, we do not know the exact hierarchy levels of all cortical areas Approximate hierarchy levels $\tilde{h}=\left(\tilde{h}\left(u_{1}\right),...,\tilde{h}\left(u_{n}\right)\right)$ for *n* cortical areas That is, we set the objective function as

$$\begin{array}{ccc} & & \underset{h}{\text{minimize}}\sum_{u_{i},u_{j}}{\left(z\left(u_{i},u_{j}\right)-\mu \left(u_{i},u_{j}\right)\right)}^{2}\\ & & \;=\underset{h}{\text{minimize}}\sum_{u_{i},u_{j}}{\left(|h\left(u_{i}\right)-h\left(u_{j}\right)|-\mu \left(u_{i},u_{j}\right)\right)}^{2}, \end{array}$$ (6)

where **h** consists of hierarchy levels of cortical areas such that **h** = (*h*(*u*_1_), …, *h*(*u*_*n*_)). The reason of considering all pairs is that the recent studies suggest the importance of role of long-distance connections in the brain [1, 24–27], and we interpret long-distance connections as paths between all cortical areas.

We fix the hierarchy levels for specific cortical areas before solving Eq (6), *e.g.*, *h*(*V*1) = 0. Then, we solve Eq (6) to compute hierarchy levels in the continuous domain, *i.e.*, *h*(*u*_*i*_) ∈ [0, *K*]. We can use an average of local minimum, since the FV91 model is also just one of the 150,000 equally plausible solution [15]. Next, we map the computed hierarchy levels to the discrete domain {0, 1, 2, …, *K*}, *e.g.*, rounding to integer.

In the experiment section, we will show that we can similarly compute hierarchy levels compared to the FV91 model with partial pairwise hierarchical relationships. We will assume that we can relatively easily infer the cortical areas which have the smallest or the largest hierarchy level. That is, we will use the partial pairwise hierarchical relationships about only some cortical areas of the minimum hierarchy level or the highest hierarchy level, and we will fix the hierarchy levels of a few cortical areas as 0 or *K* to minimize Eq (6).

### Selecting parameters

In Alg 1, we need to select two parameters *k* and *K* which are the dimension of Laplacian eigenmap and the maximum hierarchical level, respectively. We can select them by analyzing the spectral gap *γ*_*i*_ of Laplacian which are the differences between eigenvalues s.t. *γ*_*i*_ = *λ*_*i*_ − *λ*_*i*+1_, and we can compute them by using the eigen-decomposition of graph Laplacian [22]. We note that we do not use pairwise hierarchical relationships of cortical areas to compute two parameters *k* and *K*, since we need only the connectivity matrix of cortical areas whose weight are 0 or 1 to construct graph Laplacian.

**Fig 1:**
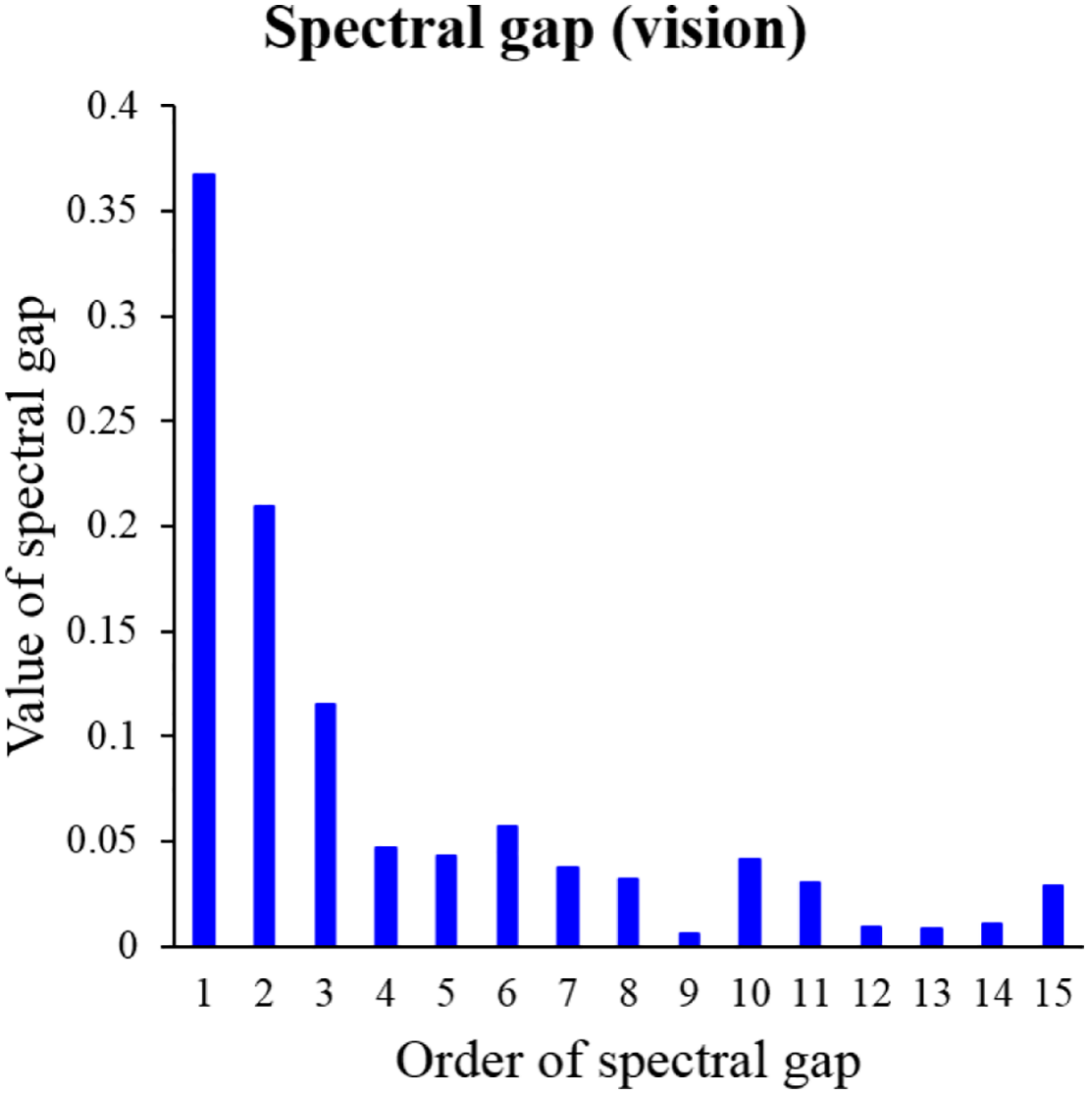
The spectral gaps of directed Laplacian of macaque vision data set. We use the spectral gaps to select parameters *k* and *K*. We compute the spectral gaps by using the eigen-decomposition of graph Laplacian, and do not use any pairwise hierarchical relationships of cortical areas.

For example, the spectral gaps of directed Laplacian **L**_*d*_ of macaque vision network are displayed in Fig 1. The 1st, 2nd, 3rd spectral gaps in Fig 1 are relatively large than others, thus we can notice that macaque vision cortical areas can be categorized as high-, medium- or low-level. However, since we want to obtain a more separated hierarchical structure, we select *k* = 4. We can also determine the maximum hierarchy level *K* by using the spectral gaps. In Fig 1, the five consecutive spectral gaps from the 4th spectral gap are relatively similar, and the 9th spectral gap is relatively very small. Thus, we can select 8 as the total number of hierarchy levels, then the maximum hierarchical level *K* is 7 when the minimum level is 0, *i.e.*, *h*(*u*_*i*_) ∈ {0, 1, 2, …, 7}. Meanwhile, *K* = 9 or *K* = 10 are also possible candidates for *K* according to the distribution of spectral gap. We note that the maximum level of the FV91 model is 9, and the maximum level of the modified FV91 model is 10. To compare the FV91 model, we set *K* = 9 in the experiments.

## Experimental results

In this section, we report the experimental results on the macaque brain data sets whose true hierarchical levels are known as FV91 model. We use the measures which are *Pearson Correlation Coefficient* (PCC), *Mean-Absolute Error* (MAE) and *Root Mean Square Error* (RMSE) for comparison.

$$\begin{array}{ccc} \text{PCC}(\tilde{x}) & = & \frac{\sum_{i}^{n}\left(x\left(u_{i}\right)-m\right)\left(\tilde{x}\left(u_{i}\right)-\tilde{m}\right)}{\sqrt{\sum_{i}^{n}{\left(x\left(u_{i}\right)-m\right)}^{2}}\sqrt{\sum_{i}^{n}{\left(\tilde{x}\left(u_{i}\right)-\tilde{m}\right)}^{2}}},\\ \text{MAE}(\tilde{x}) & = & \frac{1}{n}\sum_{i=1}^{n}|x\left(u_{i}\right)-\tilde{x}\left(u_{i}\right)|,\\ \text{RMSE}(\tilde{x}) & = & \sqrt{\frac{1}{n}\sum_{i=1}^{n}{\left(x\left(u_{i}\right)-\tilde{x}\left(u_{i}\right)\right)}^{2}}, \end{array}$$

where $\tilde{x}=(\tilde{x}\left(u_{1}\right),...,\tilde{x}\left(u_{n}\right)$, $m=\frac{1}{n}\sum_{i}^{n}x\left(u_{i}\right)$, and $\tilde{m}=\frac{1}{n}\sum_{i}^{n}\tilde{x}\left(u_{i}\right)$. The range of PCC is [−1, 1], and the optimum value of PCC is 1. The minimum of both MAE and RMSE are 0. Suppose that **x** = (*x*(*u*_1_), …, *x*(*u*_*n*_) be the hierarchy levels of FV91 model, and $\tilde{x}=(\tilde{x}\left(u_{1}\right),...,\tilde{x}\left(u_{n}\right)$ be approximate hierarchy levels computed by other methods. Then, if the approximate solution is similar with the FV91 model, then PCC will be close to 1, and MAE and RMSE will be small and close to 0.

We empirically compare our method with the FV91 model [8], hierarchical ordering with social rank [28], traditional hierarchical clustering [29], and EM-based hierarchical clustering [30]. We use directed normalized Laplacian for embedding directed graph data set. For traditional hierarchical clustering (HC), we use pdist.m, linkage.m, and cluster.m function in MATLAB with 49 different settings. We use 7 different distance metrics in pdist.m for computing pairwise distance between object pairs: Euclidean (Euclid), Standardized Euclidean (SEuclid), cosine (Cos), correlation (Corr), Chebychev (Cheb), Mahalanobis (Mah), Hamming (Ham). We also use 7 different methods in linkage.m for computing distance between clusters: average, centroid, complete, median, single, ward, weighted.

### Data sets

Let us remind that we use the connectivities of cortical areas and the partial information of pairwise hierarchical relationships for our method, and connectivities of cortical areas do not imply the pairwise hierarchical relationships. The connectivity matrix (adjacency matrix) **W** consists of 0 or 1 weights, where **W**_*i*,*j*_ = 1 means the existences of information flow from *i*-th cortical area to *j*-th cortical area, and **W**_*i*,*j*_ = 0 means the nonexistence of information flow from *i*-th cortical area to *j*-th cortical area. Since we want to analyze the hierarchical structure of macaque brain, we use two data sets which are macaque vision and somatosensory-motor data sets are provided in [8]. We note that if we want to apply our method to other data sets, we can use the connectivity obtained from fMRI, EEG, MEG, and DTI by the graph theoretical analysis of structural and functional systems [31].

For macaque vision data, we use the ‘fve32.mat’ file which includes the 32 × 32 adjacency matrix corresponding to 32 visual cortical areas and their 315 connections. The weights of connections are 0 or 1. For macaque somatosensory-motor data, we use the ‘macaque47.mat’ file which includes the 47 × 47 adjacency matrix corresponding to both vision and somatosensory-motor systems. We extract the 16 × 16 principal submatrix from the original 47 × 47 adjacency matrix to obtain the adjacency matrix of induced subgraph of 16 somatosensory-motor cortical areas. The weights of connections are also 0 or 1.

### Experiments on macaque vision data

In this section, we report the experimental results on the macaque vision data set. We use the adjacency matrix **W** which consists of only 0 or 1 values depending on the connection in the brain. The adjacency matrix **W** can not describe the pairwise hierarchical relationships such as feedforward, feedback, and lateral. Thus, we use partial pairwise hierarchical relationships in addition.

**Fig 2:**
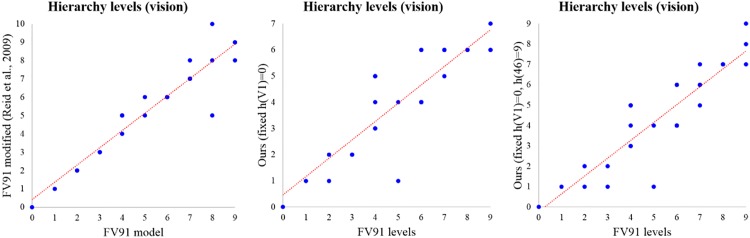
The scatter plot between the FV91 model [8] and FV91 modified model [12] for the data set of macaque brain vision (left). Comparison between the results of our method and the FV91 model on the macaque vision data set (middle and right). We used the adjacency matrix and at most 12.38% of pairwise hierarchical relationships which FV91 and FV91 modified models used. Although we used a small partial pairwise hierarchical relationships, we get similar result with the FV91 and FV91 modified models. The red line is a linear regression line.

For macaque visual cortex data set, we use the pairwise hierarchical relationships about only the V1 cortical area whose hierarchy level is the smallest, and fix its hierarchy level as 0 *i.e.*, *h*(V1) = 0. It corresponds to 5.08% = 16/315 of pairwise hierarchical relationships which FV91 model used. We run Alg 1 with *h*(V1) = 0 and Eq (6), and display the averaged results as the scatter plot in the middle of Fig 2. The scatter plot shows that hierarchy levels computed by our method are similar to the FV91 model, even though we use a very small amount of pairwise hierarchical information compared to the FV91 model and FV91 modified model.

Meanwhile, we get the TH and 46 cortical areas as the top 2 hierarchy levels in the middle of Fig 2. Hence, it would be reasonable to set *h*(TH) = *K* or *h*(46) = *K* in addition, where *K* = 9. Setting *h*(V1) = 0 and *h*(46) = *K* corresponds to 12.38% of pairwise hierarchical relationships which FV91 model used, and the experimental result is displayed as the scatter plot in the right of Fig 2. In the right scatter plot, the cortical areas of high hierarchy levels in the FV91 model have more accurate hierarchy levels compared to the scatter plot in the middle of Fig 2. Despite of using partial pairwise hierarchical relationships, the results of our method displayed in the right scatter plot have high PCC and small errors: PCC = 0.890, MAE = 1.094, and RMSE = 1.403. The result of FV91 modified model displayed in the left of Fig 2 is slightly better than ours: PCC = 0.940, MAE = 0.377, and RMSE = 0.796. However, the FV91 modified model used the entire pairwise hierarchical relationships, and it can not compute hierarchical levels of MDP and MIP regions [12]. If we consider the potential errors of MDP and MIP cortical areas in FV91 modified model, then error values of FV91 modified model may be worse.

**Fig 3:**
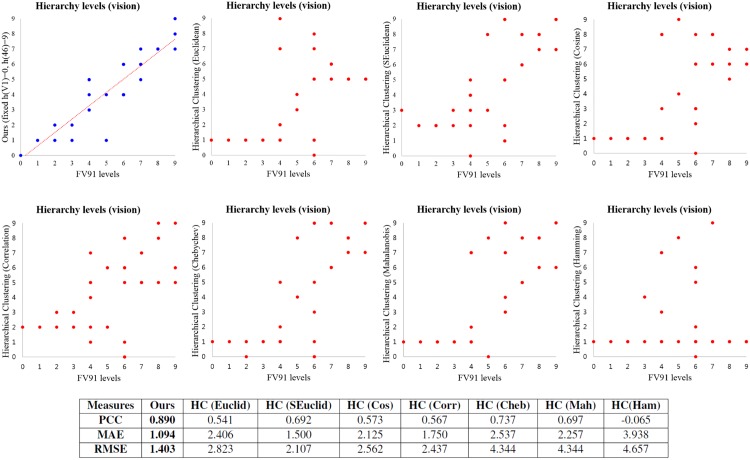
Comparison among the results of the FV91 model [8], our method and traditional hierarchical clustering methods [29] on the macaque vision data set. The performance is measured by using PCC, MAE, and RMSE. Although our method uses 12.38% of pairwise hierarchical relationships compared to the FV91 model, we get similar results to the FV91 model. In addition, the errors of our method are much smaller than the errors of traditional hierarchical clustering methods. The red line is a linear regression line.

We also compare the results of our method and various versions of traditional hierarchical clustering methods [29], and the experimental results are displayed in Fig 3. Although we select the 7 best results among 49 different settings of hierarchical clustering by considering low RMSE and high PCC, we can see that the traditional hierarchical clustering algorithms can not effectively find the hierarchical structures. Since hierarchical clustering just merges (or splits) clusters in a greedy manner by using cluster dissimilarity, it can not accurately find hierarchical structures which reflect the information flow in the brain. That is, the assigned cluster numbers from 0 to *K* for *n* nodes can not properly reflect the information flow in the brain. We can see that the cortical areas of the lowest level in the results of hierarchical clustering have hierarchical levels from 4 to 6 in the FV91 model. In addition, in the results of hierarchical clustering, we can hardly find the differences of hierarchy levels among the cortical areas which have hierarchical levels from 0 to 4 in the FV91 model. Whereas the result of our method shows an apparent hierarchical structure which is much closer to the FV91 model than the results of hierarchical clustering. There are also big differences between performances of our method and hierarchical clustering in terms of PCC, MAE, and RMSE. PCC of our method is close to the optimum 1, and is much higher than PCC of various hierarchical clustering results. Both MAE and RMSE of our method are much smaller than the results of hierarchical clustering.

**Fig 4:**
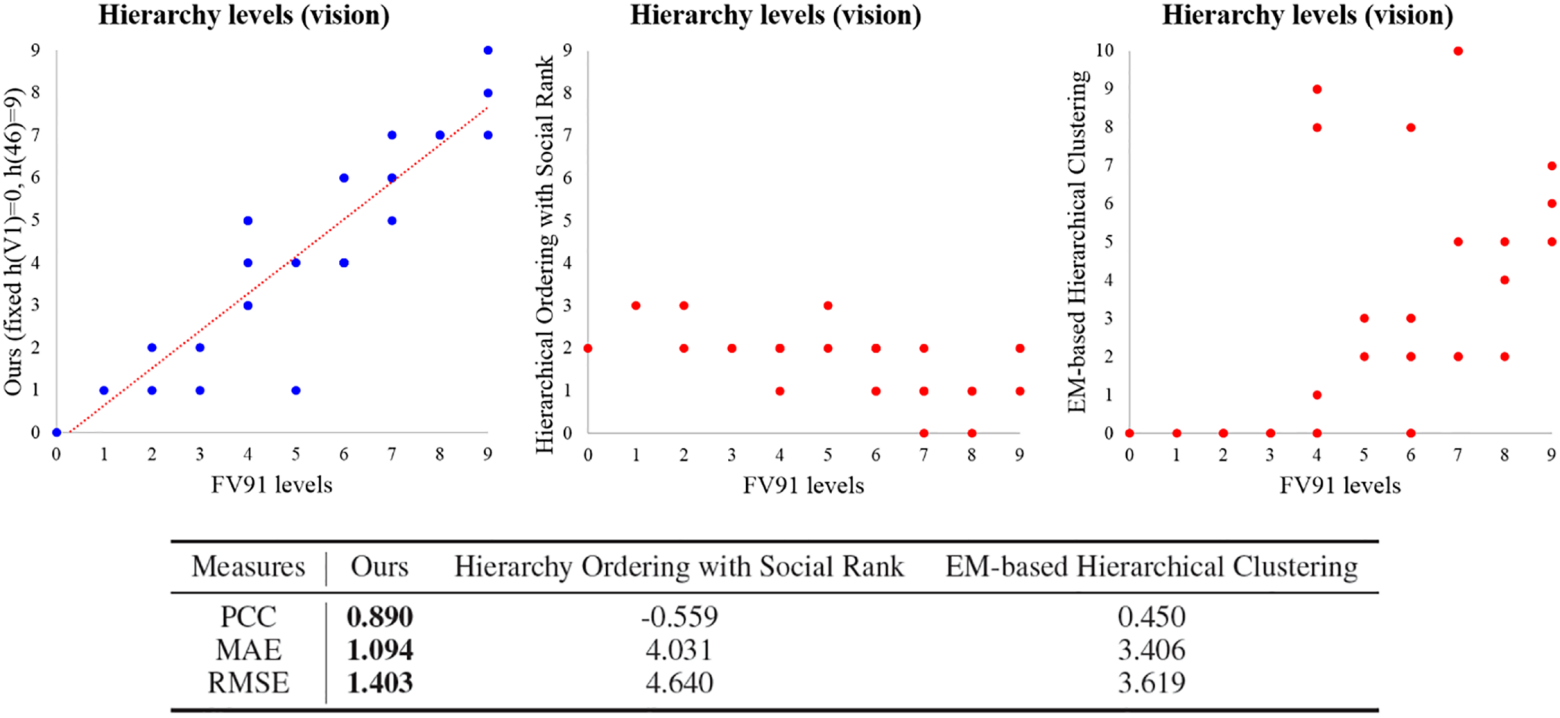
Comparison among the results of the FV91 model [8], our method, hierarchical ordering with social rank [28], and EM-based hierarchical clustering [30] on the macaque vision data set. The performance is measured by using PCC, MAE, and RMSE. The result of our method is similar to the FV91 model. In addition, the errors of our method are much smaller than the errors of hierarchical ordering with social rank, and EM-based hierarchical clustering.

Finally, we compare the results of our method, hierarchical ordering with social rank [28], and EM-based hierarchical clustering [30], and the experimental results are displayed in Fig 4. Although we select the best result of hierarchical ordering with social rank and EM-based hierarchical clustering by considering low RMSE and high PCC respectively, both methods can not effectively find the hierarchical structures. We can see that the maximum level in the result of hierarchical ordering with social rank is just 3, and we can hardly find any similar tendency between the FV91 model and the results of hierarchical ordering with social rank. We guess the reason is that the hierarchical property in the social network is quite different with the hierarchical property in the brain network. We also can not find any advantages of EM-based hierarchical clustering for hierarchical ordering of cortical areas in Fig 4. The PCC, MAE, and RMSE of EM-based hierarchical clustering are worse than the results of traditional hierarchical clustering with SEuclid distance. Thus, there are large differences among the performances of our method, hierarchical ordering with social rank, and EM-based hierarchical clustering in terms of PCC, MAE, and RMSE. PCC of our method is much higher than PCC of the two methods, and both MAE and RMSE of our method are also much smaller than the results of two methods.

### Experiments on macaque sensory-motor data

In this section, we report the experimental results on the macaque somatosensory-motor data set. We compare the FV91 model, hierarchical clustering, and our method. We again use the adjacency matrix **W** and partial pairwise hierarchical relationships.

**Fig 5:**
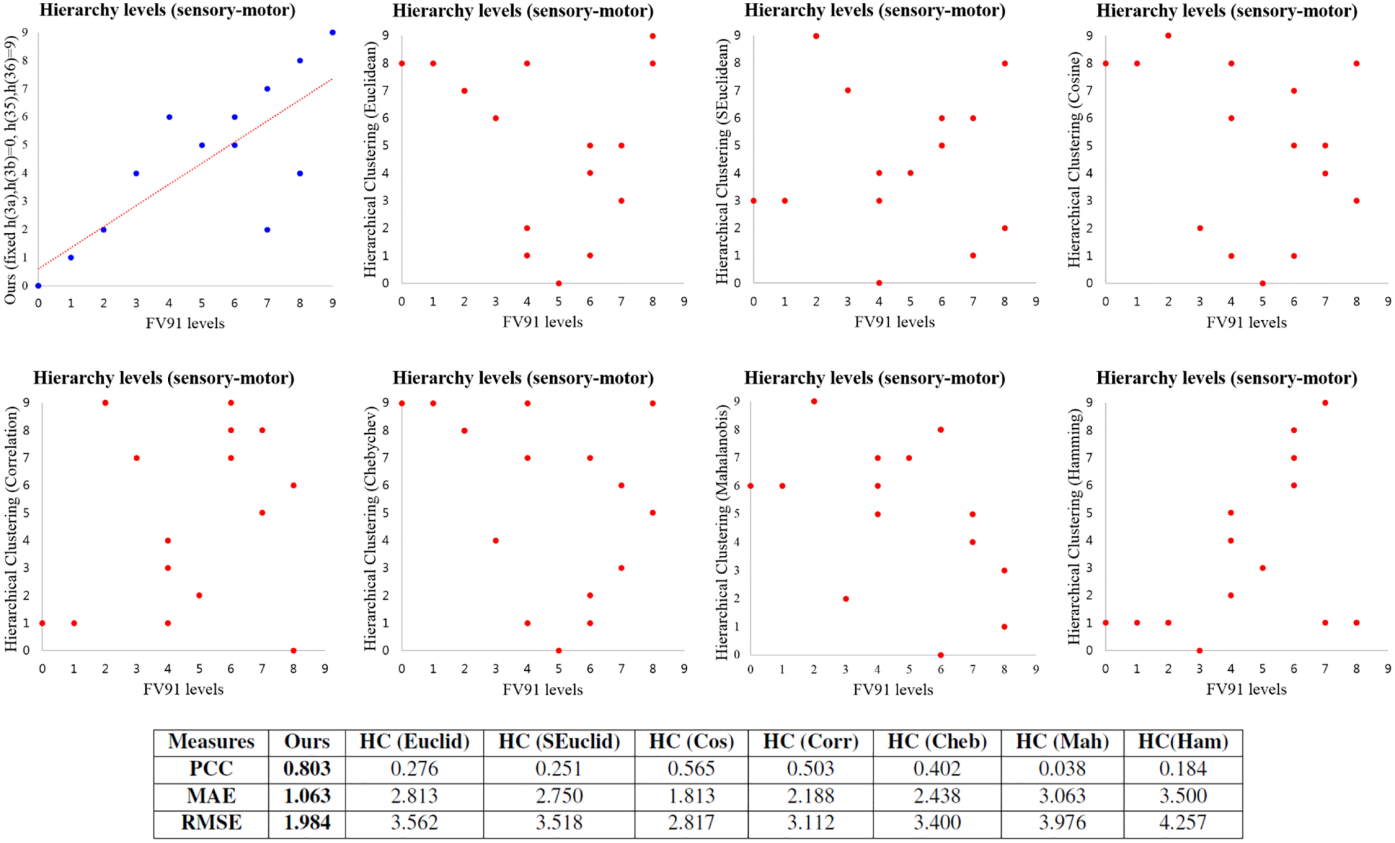
Comparison among the results of the FV91 model [8], our method and various versions of hierarchical clustering [29] on the macaque somatosensory-motor data set. The performance is measured by using PCC, MAE, and RMSE. Although our method uses 36.45% pairwise hierarchical relationships compared to the FV91 model, we get similar results to the FV91 model. In addition, the errors of our method are much smaller than the errors of hierarchical clustering. The red line is a linear regression line.

We display the experimental results of our method and the traditional hierarchical clustering methods [29] on the macaque somatosensory-motor network as the scatter plots in Fig 5. We run Alg 1 with *h*(3a), *h*(3b) = 0 and *h*(35), *h*(36) = 9 which correspond to 36.45% of pairwise hierarchical relationships which FV91 model used. The top left scatter plot in Fig 5 shows that hierarchy levels computed by our method are similar to the FV91 model, even though we use partial pairwise hierarchical relationships. We also run hierarchical clustering with 49 different settings, and select the best result for each distance metric by considering low RMSE and high PCC. We display these 7 results as the scatter plots in Fig 5. We can see that our method outperforms various hierarchical clustering approaches. The figures show that the traditional hierarchical clustering approach can not effectively find the hierarchical structures, whereas our method finds a similar hierarchical structure to the FV91 model. There are also big differences between performances of our method and hierarchical clustering approach in terms of PCC, MAE, and RMSE. PCC of our method is close to the optimum 1, and is much higher than PCC of various hierarchical clustering results. Both MAE and RMSE of our method are also much smaller than the results of hierarchical clustering.

**Fig 6:**
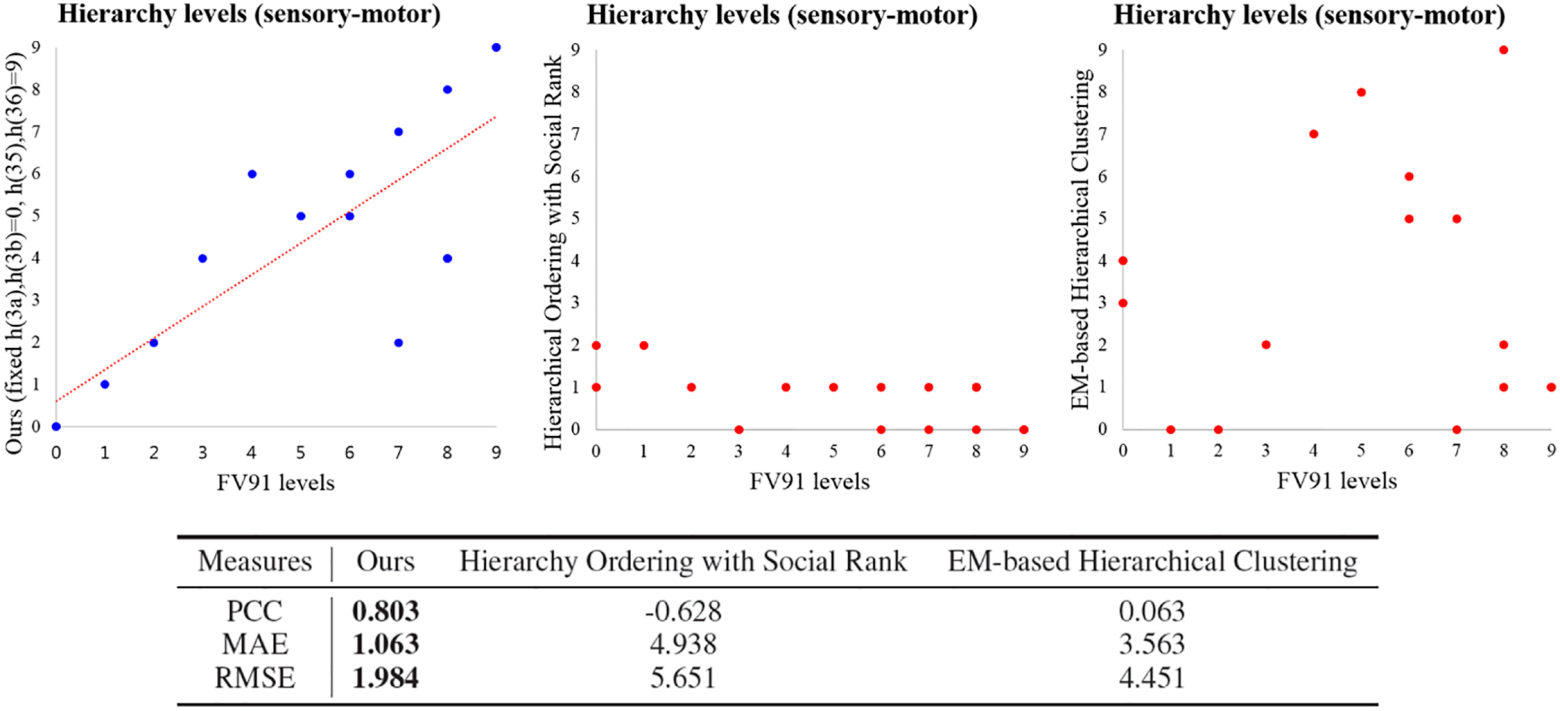
Comparison among the results of the FV91 model [8], our method, hierarchical ordering with social rank [28], and EM-based hierarchical clustering [30] on the macaque somatosensory-motor data set. The performance is measured by using PCC, MAE, and RMSE. The result of our method is similar to the FV91 model. The results of our method is superior than the results of hierarchical ordering with social rank and EM-based hierarchical clustering in terms of PCC, MAE, and RMSE.

Finally, we compare the experimental results of our method, hierarchical ordering with social rank [28], and EM-based hierarchical clustering [30] on the macaque somatosensory-motor network in Fig 6. Although we select the best result of hierarchical ordering with social rank and EM-based hierarchical clustering by considering low RMSE and high PCC respectively, both methods can not find the proper hierarchical structures. In Fig 6, we can see that the maximum level in the result of hierarchical ordering with social rank is just 2, and we can hardly find any similar tendency between the FV91 model and the results of hierarchical ordering with social rank and EM-based hierarchical clustering. PCC of our method is much higher than PCC of the two methods, and both MAE and RMSE of our method are also much smaller than the results of two methods. Especially, PCC of hierarchical ordering with social rank and EM-based hierarchical clustering are -0.628 and 0.063 respectively, and they are poor results.

## Conclusion

In this paper, we suggested a new framework that compute the hierarchy orders of cortical areas in the macaque brain by using partial pairwise hierarchical relationships. To overcome the lack of pairwise hierarchical relationships, we used a directed Laplacian eigenmap to exploit the inherent topology of the brain networks as a low-dimensional embedding, and we defined pseudo hierarchical distances for every pair of cortical areas by using the low-dimensional embedding. We computed hierarchy levels of cortical areas by minimizing the sum of squared hierarchical distance errors with the hierarchical information of few cortical areas. The experimental results showed that hierarchy levels computed by our method are similar to the FV91 model, even though we used partial pairwise hierarchical relationships. Furthermore, we showed that our method outperforms hierarchical clustering methods in terms of several error measures. Thus, we conclude that our method is quite a good compromise between the variant of FV91 models and hierarchical clustering method for computing hierarchy orders of cortical areas in the brain.
